# Interaction effects of polyfluoroalkyl substances and sex steroid hormones on asthma among children

**DOI:** 10.1038/s41598-017-01140-5

**Published:** 2017-04-18

**Authors:** Yang Zhou, Li-Wen Hu, Zhengmin (Min) Qian, Sarah Dee Geiger, Katelyn L. Parrish, Shyamali C. Dharmage, Brittany Campbell, Marjut Roponen, Pasi Jalava, Maija-Riitta Hirvonen, Joachim Heinrich, Xiao-Wen Zeng, Bo-Yi Yang, Xiao-Di Qin, Yungling Leo Lee, Guang-Hui Dong

**Affiliations:** 1 Guangzhou Key Laboratory of Environmental Pollution and Health Risk Assessment, Department of Preventive Medicine, School of Public Health, Sun Yat-sen University, Guangzhou, 510080 China; 2 Department of Epidemiology, College for Public Health and Social Justice, Saint Louis University, Saint Louis, 63104 USA; 3 School of Nursing and Health Studies, Northern Illinois University, DeKalb, IL 60115 USA; 4 Allergy and Lung Health Unit, Centre for Epidemiology and Biostatistics, Melbourne School of Population and Global Health, The University of Melbourne, Carlton, Vic 3052 Australia; 5 Department of Environmental and Biological Sciences, University of Eastern Finland, Kuopio, FI 70211 Finland; 6 Institute and Outpatient Clinic for Occupational, Social and Environmental Medicine, Clinical Center, Ludwig Maximilian University, Comprehensive Pneumology Centre Munich, German Centre for Lung Research, Ziemssenstrasse 1, 80336 Muenchen, Germany; 7 Institute of Epidemiology and Preventive Medicine, College of Public Health, National Taiwan University, Taipei, 100 Taiwan

## Abstract

To evaluate the interactions between polyfluoroalkyl substances (PFASs) and reproductive hormones and associated asthma, a total of 231 asthmatic and 225 non-asthmatic adolescents were selected from northern Taiwan in the Genetic and Biomarkers study for Childhood Asthma from 2009–2010. The interaction between PFASs and reproductive hormones on asthma was analyzed with a two-level binary logistic regression model. The results showed that, among asthmatics, PFASs were positively associated with estradiol levels and negatively associated with testosterone levels. However, only significant association was identified for PFNA and estradiol in control group. After controlling for hormone levels, associations between PFAS exposure and asthma were consistently stronger among children with higher than lower estradiol, with odds ratios (OR) for asthma ranging from 1.25 for PFOS (95% Confidence Interval [CI]: 0.90, 1.72) to 4.01 for PFDA (95% CI: 1.46, 11.06) among boys and 1.25 for PFOS (95% CI: 0.84, 1.86) to 4.16 for PFNA (95% CI: 1.36, 12.73) among girls. Notably, the interactions between estradiol and PFASs were significant for PFOS (*p* = 0.026) and PFNA (*p* = 0.043) among girls. However, testosterone significantly attenuated the association between PFOS and asthma across sex. In conclusions, our findings suggested that reproductive hormones amplify the association between PFASs and asthma among adolescents.

## Introduction

Asthma is an increasing new onset inflammatory disease that involves both intrinsic and environmental factors. Research indicates that perfluoroalkyl and polyfluoroalkyl substances (PFASs), types of persistent organic pollutants, are associated with asthma and related outcomes, including airway hyperresponsiveness, increased serum levels of immunoglobulin E (IgE), and switched type 2 T helper cell (Th2) polarization^1–4^. Anderson-Mahoney *et al*. first identified the positive association between PFOA exposure and asthma^4^. US National Health and Examination Survey (NHANES) data showed that serum PFOA concentrations are positively associated with self-reported lifetime asthma prevalence^5^.

Reproductive hormones seem to have a similar adverse effect on asthma development. Estradiol is reportedly involved in Th2 polarization, isotype switching to Ig E, mucus production, and cholinergic and histaminergic signaling in the nasal passageways^6–8^. In addition, despite inconsistent findings, a link between PFASs and sex steroid hormones has recently been widely reported^9, 10^.

To our knowledge, there are no published studies on the combined effects of PFASs and steroid hormones on asthma among adolescents, who are more developmentally vulnerable to the hormonal effects of environmental exposures than are adults. One possible reason for the dearth of literature in this area is lack of high quality study samples exposed to both PFASs and certain levels of reproductive hormones. Using data from The Genetics and Biomarkers study for Childhood Asthma (GBCA) in Taiwan, we tested the hypothesis that there were synergistic effects of the hormones and environmental pollution on asthma in children aged 10–15 years during 2009–2010 from seven public schools in Northern Taiwan.

## Methods

### Study participants

The participants in our investigation were selected from the cohort of the Genetic and Biomarkers study for Childhood Asthma (GBCA) in Taiwan during 2009–2010. Detailed information about the cohort study is available elsewhere^11^. Briefly, the cases consisted of 231 children aged 10–15 years old from two hospitals in Taipei City, China, who had been diagnosed with asthma by a physician during the previous year. Control participants consisted of 225 children without a personal or family history of asthma from seven public schools in Taipei City. The cases and controls were matched on age and sex with an average response rate of 72.0%. After providing written informed consent, the adolescents and their parents were surveyed on demographics, environmental exposures and asthma outcomes. Physical examination measures, including height, weight, waist circumference, pulmonary function, and fasting serum were collected by trained technicians. The Institutional Review Board of the National Taiwan University Hospital Research Ethics Committee approved the methods of this study, which is also compliant with the principles outlined in the Helsinki Declaration (Declaration of Helsinki, 1990).

### Key variables

Serum PFAS measurements, information about standards and reagents, sample preparation and extraction, instrumental analysis, quality assurance and quality control, and recovery experiments in the present study are detailed elsewhere^11^. In short, perfluorohexanesulfonate (PFHxS), perfluorooctane sulphonate (PFOS), perfluorooctanoic acid (PFOA), perfluorononanoic acid (PFNA) and perfluorodecanoic acid (PFDA) were measured from 0.5 mL of serum using Agilent high-performance liquid chromatography (HPLC) along with an Agilent 6410 Triple Quadrupole (QQQ) mass spectrometer (MS/MS) (Agilent, Palo Alto and Santa Clara, CA). The limit of quantification (LOQ) for PFOS, PFOA and PFNA was 0.03 ng/mL, 0.07 ng/mL for PFHxS, and 0.1 ng/mL for PFDA. All measurements were repeated and the average of the two values was the concentration of each sample used for data analysis.

We performed serum testosterone and estradiol tests to determine reproductive hormone levels at an accredited clinical laboratory. We measured reproductive hormones in serum by immunoluminometric assay with an Architect random access assay system (Abbott Diagnostics, AbbottPark, IL). The limit of detection (LOD) for estradiol and testosterone were 18 pmol/L and 0.23 nmol/L, respectively. We used 50 μL in analysis for testosterone and estradiol. The intra-assay and inter-assay coefficients of variation of these measurements were all below 10%, and 15%, respectively. Of the blood samples, 67% were measured within 12 hours, and 33% between 12 and 17 hours within collection.

### Statistical analysis

We tested the data for normality (Q-Q plots) and homogeneity (Bartlett’s test for unequal variances), making appropriate transformations when necessary. Normal and homogenous continuous variables are given as either the mean ± SD, or the median and quartile 1 (Q1)-quartile 3 (Q3). We used a *t*-test to compare the normally distributed continuous variables with asthma status, and the Wilcoxon rank-sum test to compare PFAS concentrations with asthma status due to skewed PFAS distributions. Sex hormones were natural log transformed for analyses. We tested relationships between categorical variables using contingency tables and the Chi-square test, and used multiple general linear models to estimate the relationship between reproductive hormone levels and a single PFAS exposure variable (adjusting for covariates).

We also employed multivariate linear regression analysis to explore the relationships between PFASs and reproductive hormones. Finally, to investigate the effect of hormone and PFAS interaction on asthma, we conducted a two-level binary logistic regression model with children being the first-level unit and exposure being the second-level unit, and adjusted odds ratios (ORs) with 95% confidence intervals (95% CIs) were calculated. At the child level, we modeled the logit of the prevalence of asthma by hormone level categories and other covariates. At the exposure level, the random coefficients were regressed on the pollutant level to explain the variations of the different level intercepts and coefficients. In the two-level binary logistic regression model, five PFASs—PFOA, PFOS, PFDA, PFHxS and PFNA—were considered key exposure variables. Covariates included were estradiol (lower than median, higher than median), age (years), sex (male, female), body mass index (BMI; kg/m^2^), parental education (less than and higher than high school), environmental tobacco smoke exposure (ETS, collected the smoking status of each participant’s adult household members and regular household visitors from current and past time), physical activity (at least 1 hour per day outside of physical education class), and month of survey (July-September, November-December). Statistical analyses were performed by the GLIMMIX procedure using SAS software (version 9.4, SAS Institute Inc., Cary, NC, USA).

## Results

Table 1 shows descriptive characteristics of the study population. Compared to children without asthma, male and younger children tended to have asthmatic symptoms (Table 1). In addition, levels of serum testosterone and estradiol were significantly higher among asthmatic children (*p* < 0.01). All median serum PFAS levels were significantly higher among asthmatic children than non-asthmatic children (*p* < 0.01). PFOS was higher in concentration than other PFASs, with a median of 28.91 ng/ml in non-asthmatics and 33.94 ng/ml in asthmatics.

**Table 1:** Characteristics of the study population (n = 456).

Characteristic	With asthma	Without asthma	*p*-Value
	(n = 231)	(n = 225)	
Age (years)	12.85 ± 1.67	13.61 ± 0.73	<0.001
Height (cm)	156.62 ± 10.43	159.83 ± 7.00	<0.001
Weight (kg)	49.83 ± 13.29	52.50 ± 13.21	0.033
BMI (kg/m^2^)	20.07 ± 3.89	20.40 ± 4.12	0.379
Sex^*^			
Boy	158 (60.77)	102 (39.23)	<0.001
Girl	73 (37.24)	123 (62.76)	
Regular exercise^*^			
No	38 (41.76)	53 (58.24)	0.058
Yes	193 (52.88)	172 (47.12)	
Parental education^*^			
<high school	90 (51.14)	86 (48.86)	0.871
≥high school	141 (50.36)	139 (49.64)	
Environmental tobacco smoke (ETS) exposure^*^			
No	138 (59.74)	93 (40.26)	<0.001
Yes	93 (41.33)	132 (58.67)	
Month of survey^*^			
July-September	106 (40.46)	156 (59.54)	<0.001
November-December	125 (64.43)	69 (35.57)	
PFOS (ng/mL)^†^	33.94 (19.59–61.10)	28.91 (14.06–42.02)	0.002
PFOA (ng/mL)^†^	1.16 (0.48–2.16)	0.52 (0.44–1.27)	<0.001
PFDA (ng/mL)^†^	1.14 (0.89–1.48)	0.95 (0.76–1.15)	<0.001
PFHxS (ng/mL)^†^	2.47 (1.25–4.26)	1.32 (0.59–2.79)	<0.001
PFNA (ng/mL)^†^	1.00 (0.73–1.28)	0.83 (0.64–1.05)	<0.001
Testosterone (nmol/L) (median)	11.47 ± 7.72 (13.90)	9.15 ± 8.99 (2.94)	0.003
Estradiol (pmol/L) (median)	178.67 ± 70.50 (166.72)	151.47 ± 65.05 (140.55)	<0.001

Values are mean ± SD.

^*^Values are number (%).

^†^Values are median (Q1–Q3).

Table 2 shows the results of multivariate linear regression analysis evaluating associations between PFASs and sex hormones, stratified by asthma category. Among participants without asthma, the only significant association was between PFNA and testosterone (β = 0.206; 95% CI: 0.002, 0.411). However, among asthmatics, four of five PFASs were significantly, positively associated with estradiol: PFOS (β = 0.001; 95% CI: 0.000, 0.003), PFOA (β = 0.054; 95% CI: 0.019, 0.089), PFDA (β = 0.096; 95% CI: 0.018, 0.174) and PFNA (β = 0.142; 95% CI: 0.050, 0.234). In addition, two of five PFASs were significantly, negatively associated with testosterone: PFOS (β = −0.004; 95% CI: −0.005, −0.003) and PFOA (β = −0.036; 95% CI: −0.072, −0.001).

**Table 2:** Association between PFASs (ng/mL) and ln-(sex hormones) using multivariate linear regression analysis by asthma category.

	Without asthma(n = 225)		With asthma(n = 231)	
	Testosterone (nmol/L)	Estradiol (pmol/L)	Testosterone (nmol/L)	Estradiol (pmol/L)
PFOS	−0.002	0.002	**−0.004**	**0.001**
	(−0.008, 0.003)	(−0.006, 0.004)	**(−0.005, −0.003)**	**(0.000, 0.003)**
PFOA	0.064	0.057	**−0.036**	**0.054**
	(−0.090, 0.219)	(−0.011, 0.125)	**(−0.072, −0.001)**	**(0.019, 0.089)**
PFDA	−0.194	0.053	−0.042	**0.096**
	(−0.484, 0.097)	(−0.076, 0.183)	(−0.122, 0.037)	**(0.018, 0.174)**
PFHxS	−0.004	0.025	−0.001	0.001
	(−0.073, 0.065)	(−0.006, 0.056)	(−0.006, 0.003)	(−0.004, 0.005)
PFNA	−0.341	**0.206**	−0.025	**0.142**
	(−0.804, 0.121)	**(0.002, 0.411)**	(−0.119, 0.070)	**(0.050, 0.234)**

All models are adjusted for age, sex, body mass index, parental education, environmental tobacco smoke exposure, physical activity, and month of survey.

Coefficient (β) and 95 confidence intervals (CI) represent the change in ln-(sex hormone) levels for each 1 ng/mL increase in PFAS concentration.

Tables 3 and 4 show interactions between PFASs and reproductive hormones, and their association with asthma. The association between individual PFAS exposure and asthma prevalence was consistently stronger among children with higher estradiol levels, with odds ratios (ORs) ranging from 1.06 to 1.69 for lower estradiol boys, 1.25 to 4.01 for higher estradiol boys, 0.65 to 3.54 for lower estradiol girls, and 1.25 to 4.16 for higher estradiol girls. Specifically, estradiol significantly enhanced the association of PFOS, PFDA and PFNA with asthma among adolescents (*p* < 0.10) (Table 4). However, testosterone modified the associations of only PFOS and asthma significantly, across sex (*p* < 0.05) (Table 3).

**Table 3:** Interaction effects of PFASs (ng/mL) and testosterone category on asthma, by sex^ab^.

	Low testosterone	High testosterone	Interaction
	OR (95% CI)^b^	OR (95% CI) ^b^	P-value
**Boys**			
PFOS	2.54	1.04	**0.005**
	(1.40, 4.60)	(0.87, 1.25)	
PFOA	2.82	2.42	0.660
	(1.60, 4.97)	(1.47, 3.99)	
PFDA	1.71	3.16	0.339
	(0.75, 3.90)	(1.21, 8.25)	
PFHxS	2.12	1.43	0.165
	(1.34, 3.35)	(0.99, 2.07)	
PFNA	2.05	4.52	0.272
	(0.75, 5.58)	(1.59, 12.84)	
**Girls**			
PFOS	1.32	0.58	**0.010**
	(0.88, 1.99)	(0.36, 0.93)	
PFOA	2.88	3.16	0.842
	(1.39, 5.97)	(1.47, 6.78)	
PFDA	1.24	1.37	0.845
	(0.60, 2.56)	(0.63, 3.02)	
PFHxS	1.62	2.27	0.334
	(1.08, 2.45)	(1.29, 3.99)	
PFNA	1.15	4.25	0.121
	(0.39, 3.37)	(1.20, 15.03)	

^a^low vs high grouped according to median testosterone level.

^b^All models adjusted for age, sex, body mass index, parental education, environmental tobacco smoke exposure, physical activity, and month of survey.

**Table 4:** Interaction effects of PFASs (ng/mL) and estradiol category on asthma, by sex^ab^.

	Low estradiol	High estradiol	Interaction
	OR (95% CI) ^b^	OR (95% CI) ^b^	P-value
**Boys**			
PFOS	1.06	1.25	0.407
	(0.87, 1.30)	(0.90, 1.72)	
PFOA	1.85	2.93	0.214
	(1.12, 3.06)	(1.64, 5.24)	
PFDA	1.21	4.01	**0.060**
	(0.60, 2.46)	(1.46, 11.06)	
PFHxS	1.47	1.62	0.741
	(1.00, 2.15)	(1.01, 2.60)	
PFNA	1.69	3.79	0.289
	(0.57, 5.00)	(1.31, 10.96)	
**Girls**			
PFOS	0.65	1.25	**0.026**
	(0.42, 0.99)	(0.84, 1.86)	
PFOA	3.54	2.56	0.486
	(1.61, 7.79)	(1.27, 5.12)	
PFDA	0.76	1.78	0.176
	(0.27, 2.20)	(0.94, 3.35)	
PFHxS	2.39	1.65	0.281
	(1.39, 4.12)	(1.07, 2.55)	
PFNA	0.82	4.16	**0.043**
	(0.26, 2.61)	(1.36, 12.73)	

^a^low vs high grouped according to median estradiol level.

^b^All models adjusted for age, sex, body mass index, parental education, environmental tobacco smoke exposure, physical activity, and month of survey.

## Discussion

This case-control study among 456 Taiwanese children aged 10–15 years is the first, to our knowledge, to explore the interaction between PFASs and reproductive hormones on asthma outcomes. Though inevitable confounding or other sources of bias cannot be ruled out, we found more significant associations between PFASs and hormones among asthmatic than among non-asthmatic children. We also found that the associations between exposure to each PFAS and the prevalence of asthma was consistently stronger among higher estradiol level children than among children with lower estradiol, with ORs estimated ranging from 1.06 to 1.69 for low estradiol boys, 1.25 to 4.01 for higher estradiol boys, 0.65 to 3.54 for low estradiol girls, and 1.25 to 4.16 for high estradiol girls. These results suggested estradiol levels amplify the association between PFAS exposure and asthma.

Literature on this topic is sparse; a PubMed/Medline search yielded, no studies on the interaction between PFASs and reproductive hormones, and asthma. However, relevant studies about the relationship between both PFASs and asthma, and sex steroids and asthma are well documented^2, 4, 5, 11^.

For the association between PFASs and asthma, experimental research has reported a potential positive association. In a murine model of asthma, Fairley *et al*. reported a dose-response relationship between PFOA concentration and the OVA-specific airway hyper reactivity response, as well as a pleiotropic cell response characterized by eosinophilia and mucin production^2^. Another *in vitro* experiment showed PFOA increased both histamine release and expression of pro-inflammatory cytokines, which induce mast cell-derived allergic inflammatory reactions^3^. In addition, one of our own previous studies showed PFOS exposure increased IL-4 and IL-10 but decreased IL-2 and IFN-γ secretion, meaning hosts’ immunity favors a T(H)2-like state^1, 12^.

Evidence from epidemiologic studies also supports associations between environmental PFASs and asthma among adolescents. In a cross-sectional study of 566 community-dwelling participants with prolonged PFOA exposure through contaminated drinking water, Anderson-Mahoney *et al*. found an association between PFOA exposure and asthma^4^. Specifically, asthma prevalence increased among participants exposed to PFOA compared to the general U.S. population (SPR = 1.82, 95% CI: 1.47, 2.25). Our present study also tested the associations between PFAS concentrations and asthma and observed positive associations among Taiwanese children, with the exception of PFOS, which was positively associated with asthma with adjusted OR among those with the highest versus lowest quartile of PFASs exposure ranged from 1.81 (95% CI: 1.02, 3.23) for PFDoA to 4.05 (95% CI: 2.21, 7.42) for PFOA^11^. Recently, Smit *et al*. used meta-analysis to explore associations between contaminants measured in serum of pregnant women and both asthma and eczema among school-age children in the INUENDO birth cohort^13^. They applied principal components analysis and found the PC5 score, dominated by PFOA, was negatively associated with current wheezing (OR = 0.64; 95% CI: 0.41–0.99) in 612 samples from Ukraine. This inconsistent finding may result from the interaction between PFASs and reproductive hormones. Further, adolescents may be more susceptible to exogenous and endogenous hormone variation than their adult counterparts. The results showed higher PFAS concentrations decreased testosterone and increased estradiol in adolescents significantly, but no such significant association was identified among their adult counterparts association^14, 15^.

There is also clear evidence that reproductive hormones influence asthma development and severity. Reproductive hormones have been seen to affect cholinergic and histaminergic signaling in the nasal passageways, dissociation of endothelial NO synthase (an indicator of airway inflammation), prostaglandin synthesis and cGMP modulation of airway smooth muscle, and changes in immune response modulation^8^. In addition, research shows that estradiol or progesterone may skew the immune response toward Th2^6^, isotype switching to immunoglobulin E^7^, and mast cell degranulation^16^ in experimental studies. However, testosterone either tilts the response to Th1 or suppresses inflammation^17, 18^. Giro´n-Gonza´lez reported higher secretion of Th1 cytokines IFN-γ and IL-2 in lymphocytes stimulated with phytohemagglutinin from males than females^17^. A 2012 epidemiologic study confirmed higher prevalence of asthma among males than females prior to the onset of puberty^8^. However, female post-puberty asthma incidence is double that of males, and menopause is associated with accelerated lung function decline and increased new-onset asthma^19, 20^. Furthermore, menarche is also an important factor in women developing asthma^21^. Macsali reported women with early menarche (before age 10 years) have lower lung function and more asthma compared with women with later menarche. Thus, all the above results suggest that estradiol is a risk factor for asthma development, whereas testosterone is protective.

The way in which PFAS and sex hormones modulate the underlying biological mechanisms remains unclear. One possible explanation is that systemic inflammation, a widely known effect of PFASs, dictates emergence and development of asthma. It is thought that PFASs have an effect on most steps of allergic sensitization: type 2 T helper cell (Th2) polarization^1^, isotype switching to immunoglobulin E^1, 2^, and mast cell degranulation^3^. TH1/TH2 polarization toward TH2 responses is common among atopy diseases^22^, and IgE is involved in mediating type 1 hypersensitivity reactions, including asthma^23^. In addition, mast cells could release the major mediators of acute hypersensitivity (e.g. histamine, cysteinyl leukotrienes), an obligatory event in allergic reactions^24^.

An increasing number of studies have shown that endogenous estradiol also can skew the immune response in favor of Th2^6^, isotype switching to immunoglobulin E^7^, and mast cell degranulation^16^, and that these changes can result in bronchial epithelium inflammation, fibrosis and airway constriction, and ultimately asthma. As PFASs and estradiol are both associated with increasing inflammation, and asthma is an inflammatory disease of the airways that involves both intrinsic and environmental factors that promote inflammation in bronchoalveolar-lavage fluid or pulmonary secretions^25^, we hypothesize that adolescents with higher estradiol would be more susceptible to the inflammatory effects of PFAS exposure, leading to elevated asthma prevalence.

Finally, PFASs, as well as a variety of endocrine disrupting chemicals, have reportedly affected estrogen synthesis, release, and function. Previous studies reported that PFASs affect enzyme synthesis and steroidogenicity, combine with estrogen receptors (ER) easily, and disrupt the reproductive axis^26–29^. Du *et al*. reported PFOA and PFOS could enhance ER-transactivation in the presence of 17β-estradiol while having direct binding affinity for ER in the absence of 17β-estradiol^26, 27^. ER has been demonstrated on human bronchial epithelial cells and binds respective hormones that crosstalk with an array of cellular signaling networks^30^. Thus, PFASs and estradiol may have a synergistic effect on the underpinnings of asthma development.

One interesting finding shows that the significant interaction on asthma was found for sex steroid hormones with PFOS, but not with PFOA, which indicates that PFOS is more likely to produce joint effect with hormones on atopic diseases. The reasons differences between PFOS and PFOA are not clear but may be associated with the PFASs concentrations. In present study, the serum concentrations of PFOS were 33.94 ng/mL in asthmatics and 28.91 ng/mL in children without asthma, which were about 30-fold higher than the levels of PFOA (1.16 ng/mL in asthmatics and 0.52 ng/mL in children without asthma, respectively). In addition, some studies also reported the differences of toxicological response *in vivo* exposed to PFOS and PFOA. Recently, Kang *et al*. investigated the endocrine-related effects of PFOA and PFOS using *in vitro* estrogen receptor (ER) and androgen receptor (AR) transactivation assays and steroidogenesis assay^31^, and the results showed that the gene expression of *cyp11b2* related to corticoid biosynthesis was significantly increased by only PFOS. It is known that CYP11B2 is an enzyme involved in the biosynthesis of aldosterone, which play a key role in the development of atopy diseases including asthma^32^. However, as the agonists for peroxisome proliferator-activated receptors (PPARs) signaling pathway^33^, Takacs and Abbott conducted a study to characterize the differential activation of mouse and human PPAR-α, PPAR-β, and PPAR-γ, and the results indicated that PFOA had more trans-activity than PFOS with both the mouse and human PPAR isoforms^34^. Based upon these types outcomes in the above-cited studies, future studies will need to be undertaken to explore the mechanism underlying the difference effects between PFOS and PFOA.

Although this study provides new information on the influence of PFASs’ and reproductive hormones’ complex interaction effects on asthma among children and adolescents, there are some limitations that should be addressed. First, establishing causal relationships between PFAS exposure and asthma outcomes is not possible due to the cross-sectional nature of the study. Second, the asthma cases and controls were recruited from hospitals and schools, respectively, making selection bias and uncontrolled confounding possible.

In the current study, we demonstrated the additive effects of PFAS and reproductive hormones on asthma in puberty, an important developmental period regarding asthma. As previously noted, males experience more cases of asthma until the onset of puberty, when the sex distribution of asthma is reversed due to the huge fluctuations in sex hormone levels at the onset of puberty. Thus, it is important to research the pathogenesis of and risk/protective factors for asthmatic children during this sensitive developmental period.

In conclusion, our results indicate that an increase in PFASs concentration was associated with asthma among adolescents, and this association seems stronger in those with higher estradiol levels. Our study is the first step toward understanding how PFAS exposure and hormones interact to affect asthma. The results of this research are consistent with the hypothesis that the effects of both environmental and endogenous estrogens together will determine asthma status.
